# Performance Comparison of a Duplex Implementation of the CDC EUA 2019-nCoV Assay with the Seegene Allplex-SARS-CoV-2 Assay for the Detection of SARS-CoV-2 in Nasopharyngeal Swab Samples

**DOI:** 10.3390/mps5050073

**Published:** 2022-09-21

**Authors:** Karen Marcela Jiménez, Dora Janeth Fonseca-Mendoza, Adrien Morel, Oscar Ortega-Recalde, Nora Constanza Contreras Bravo, Camilo Andres Velandia-Piedrahita, Adriana Steevens, Luisa Daniela Caldas, Juan Pablo Ribón, Martha Sánchez, Carlos Martin Restrepo, Rodrigo Cabrera

**Affiliations:** 1 Laboratorio de Biología Molecular y Pruebas Diagnósticas de Alta Complejidad, Fundación Cardioinfantil-Instituto de Cardiología, Bogotá 110131, Colombia; 2 Center for Research in Genetics and Genomics—CIGGUR, GENIUROS Research Group, School of Medicine and Health Sciences, Universidad del Rosario, Bogotá 111221, Colombia; 3 Institute of Translational Medicine (IMT), School of Medicine and Health Sciences, Universidad del Rosario, Bogotá 111221, Colombia

**Keywords:** COVID-19, molecular diagnostic, SARS-CoV-2, real-time PCR

## Abstract

RT-PCR tests have become the gold standard for detecting the SARS-CoV-2 virus in the context of the COVID-19 pandemic. Because of the extreme number of cases in periodic waves of infection, there is a severe financial and logistical strain on diagnostic laboratories. For this reason, alternative implementations and validations of academic protocols that employ the lowest cost and the most widely available equipment and reagents found in different regions are essential. In this study, we report an alternative implementation of the EUA 2019-nCoV CDC assay which uses a previously characterized duplex PCR reaction for the N1 and RNAse P target regions and an additional uniplex reaction for the N2 target region. Taking advantage of the Abbott m2000 Sample Preparation System and NEB Luna Universal Probe One-Step RT-qPCR kit, some of the most widely available and inexpensive nucleic acid extraction and amplification platforms, this modified test shows state-of-the-art analytical and clinical sensitivities and specificities when compared with the Seegene Allplex-SARS-CoV-2 assay. This implementation has the potential to be verified and implemented by diagnostic laboratories around the world to guarantee low-cost RT-PCR tests that can take advantage of widely available equipment and reagents.

## 1. Introduction

On 30 January 2020, the World Health Organization (WHO) declared infection with the SARS-CoV-2 virus as a public health emergency of international concern after it rapidly spread throughout the world, causing the COVID-19 pandemic [1]. The first reports of the virus were reported in Wuhan, China in December 2019, and more than 340 million confirmed cases and more than 5 million deaths have been reported worldwide since. Global waves of novel variants such as Delta and Omicron have made the pandemic a persistent challenge.

To counteract this health emergency through clinical and public health interventions, massive testing has emerged as one of the main strategies to track the spread of the virus, allowing for the prompt and adequate in vitro diagnosis of SARS-CoV-2. To achieve this goal, it is important to identify and validate low-cost, high-throughput protocols that can be implemented quickly with adequate operational characteristics.

The Centers for Disease Control and Prevention of the United States (CDC) designed the EUA 2019-nCoV CDC assay for the detection and identification of SARS-CoV-2 in the United States. This test detects two different regions of the N gene (Nucleocapsid gene), and the human RNase P gene as an internal control, by means of three independent real-time PCR reactions [2]. Due to the health emergency, the CDC began sending these kits to different laboratories in the United States for the diagnosis of COVID-19. However, several of these laboratories reported positive amplification in negative controls of this kit, possibly as a consequence of contamination in the reagents and a design flaw in a target region, which has since been excluded from the test [3,4]. This problem was promptly solved by the CDC to continue its use as a routine diagnostic method in the United States [5,6]. Different implementations of the assay using the same primer and probe sequences have been widely used for the detection of SARS-CoV2 [2,7,8,9,10]. However, the reagent costs for many of these implementations is still relatively high considering the large number of tests required to efficiently control the spread of the disease. Furthermore, the original validation of this test did not evaluate some of the most widely available equipment and reagents for nucleic acid extraction and amplification.

In this study, we report the validation of the CDC protocol using the widely available Abbott m2000 Sample Preparation System (Abbot, Abbott Park, IL, USA) and NEB Luna Universal Probe One-Step RT-qPCR kit (New England Biolabs, Ipswich, MA, USA). This test determines the presence of SARS-CoV-2 RNA by means of two real-time PCR assays that are performed simultaneously. The assay uses a previously characterized duplex PCR reaction for the N1 and RNAse P target regions and an additional uniplex reaction for the N2 target region [11]. This setup allows for the use of the primers and probes for the N1 and N2 target regions, as reported in the EUA 2019-nCoV CDC assay, without modifying their reported chemistry. The reagents used are compatible with both manual and automated extraction procedures. The analytical Limit of Detection (LoD), cross-reactivity and clinical performance of the assay were evaluated, with satisfactory results.

## 2. Materials and Methods

### 2.1. Sample Processing

Excess samples from diagnostic testing for SARS-CoV-2 were anonymized and retested using the procedure described herein. Ethical review and approval were waived for this study because the results presented involve leftover anonymized samples intended for diagnostic test validation. Guidelines for sample management during the SARS-CoV-2 (COVID-19) pandemic in Colombia were followed, as determined by the Ministry of Health and Social Protection in October 2020 (PSPS02) [12]. Briefly, nasopharyngeal semisynthetic swabs were used to collect the samples. The swabs were deposited into a collection tube which contained approximately 1.5 mL of albumin-based viral transport media (VTM) and transported to the Molecular Biology Laboratory (Fundación Cardioinfantil Instituto de Cardiología, Bogotá, Colombia), where they were frozen at −80 °C until processing [12]. The samples were processed after thawing at 4 °C overnight. During sample collection, patients were allowed to decide whether their leftover samples would be available for method validation through a clinical procedure informed consent form, as required by local guidelines [13].

### 2.2. Amplification Design

Nucleic acids were extracted using the Abbott m2000sp automated Sample Preparation System and Abbott mSample Preparation SystemDNA (Abbott, Abbott Park, IL, USA) from 800 µL of the nasopharyngeal swab sample, following the manufacturer’s instructions. This Abbott mSample Preparation SystemDNA employs a protocol based on lysis with guanidine isothiocyanate and magnetic microparticles to extract total nucleic acids (both DNA and RNA). This principle is similar to the commercially available RNA extraction kits and procedures that have been qualified, validated or accepted for use with the EUA 2019-nCoV CDC assay. However, the higher sample and lower elution volumes of the Abbott mSample Preparation SystemDNA are likely to lead to higher concentrations of nucleic acids from similar samples, assuming RNA capture efficiencies similar to those of other methods (Supplemental Table S1).

Subsequently, the extracted RNA was inverse transcribed into complementary DNA (cDNA) and amplified using the Luna Universal Probe One-Step RT-qPCR kit (New England Biolabs, Ipswich, MA, USA) [14]. A master mix was prepared for assay 1 by adding 12.5 ul Luna Universal Probe One-Step Reaction Mix (2×), 1.25 µL Luna WarmStart RT Enzyme Mix (20×), 0.1 µL 2019-nCoV-N1 of fwd and rev primers, 0.05 µL of 2019-nCoV-N1 Probe, 0.0375 µL of Human RNase P fwd and rev primers, 0.05 µL Human RNAse P Probe and 0.875 µL of molecular grade water. A master mix was prepared for assay 2 by adding 12.5 µL Luna Universal Probe One-Step Reaction Mix (2×), 1.25 µL Luna WarmStart RT Enzyme Mix (20×), 0.1 µL 2019-nCoV-N2 of fwd and rev primers, 0.05 µL of 2019-nCoV-N2 Probe and 1 µL of molecular grade water in each amplification reaction. A total of 15 µL of master mix was added to 10 µL of eluate. The duplex N1/Rnase P assay has previously been shown to have comparable sensitivity to the original EUA 2019-nCoV CDC using chemistries identical to the ones used here [11]. The primer and probe sequences are shown in Table 1. This master mix was thawed a maximum of two times without observing changes in performance after two thaws, since the Cts (cycle thresholds) of the positive controls of the first and second thaw were similar (data not shown). The amplification protocol consisted of a reverse transcription step of 10 min at 55 °C, followed by one initial denaturation step of 3 min at 95 °C and 40 cycles of 10 s at 95 °C and 30 s at 55 °C. This program was run in a CFX96 Touch real-time PCR system (BioRad, Hercules, CA, USA). The detection channels used were FAM (N1 and N2 targets) and Quasar 670 (RP gene), with fluorescence measured at the end of each cycle of amplification. To evaluate the performance of the Luna Universal Probe One-Step RT-qPCR kit in comparison with the different enzyme master mixes evaluated for the EUA 2019-nCoV CDC assay, we performed amplification reactions of the N1 and N2 targets using the RGTM10169 SARS-CoV-2 Research Grade Test Material from the National Institute of Standards and Technology (NIST, Gaithersburg, MD, USA), as described, without carrying out the RNA extraction step.

### 2.3. Test Performance Evaluation

#### 2.3.1. Determination of the Analytical Limit of Detection (LoD) of the Test

The LoD was defined as the lowest detectable concentration of SAR-CoV-2 required to obtain a positive result in 95% of replicates. To determine this parameter, serial dilutions of 400, 200, 100, 50 and 25 copies/mL of the AccuPlex™ SARS-CoV-2 Reference Material Kit (SeraCare Life Sciences, Milford, MA, USA) were analyzed in VTM. Four independent tests were performed, under reproducible conditions, to obtain a total of 20 replicates per dilution. To calculate the LoD, a Probit regression was carried out using the statistical software MedCalc (MedCalc Software Ltd., Ostend, Belgium) and SPSS (version 21, SPSS, Inc., New York, NY, USA). The results from the analysis with SPSS are shown. Inter-assay reproducibility testing was performed by independently (*n* = 79) evaluating the Ct value of the assay positive control, which consistently scores slightly lower (approximately 1 Ct unit below the LoD; mean 36.17 for N1 and 37.62 for N2) than the limit of detection of the assay. Intra-assay reproducibility testing was performed by evaluating the Coefficient of Variance of 21 assays with 5 replicates each using spiked simulated samples of the AccuPlex™ SARS-CoV-2 Reference Material at 400, 200, 100, 50 and 25 copies/mL. Only assays where the target was detectable in the five replicates were used: *n* = 6 for 400 copies/mL for both targets, *n* = 6 for 200 copies/mL for both targets, *n* = 5 and *n* = 6 for 100 copies/mL for N1 and N2, respectively, *n* = 4 and *n* = 1 for 50 copies/mL for N1 and N2, respectively, and *n* = 1 for 25 copies/mL for both targets.

#### 2.3.2. Verification of the Cross-Reactivity of the Test

The cross-reactivity analysis of the primers and probes used in the present test was described in the CDC validation protocol (2). In this analysis, no cross-reactivity was found when analyzing isolates, clinical samples and bacterial cultures of different respiratory pathogens. In total, 20 viruses and 2 bacterial species, including coronaviruses 229E, OC43, HKU1, NL63, coronavirus-SARS and coronavirus-MERS; influenza A and B; adenovirus; bocavirus; enterovirus D68; human metapneumovirus; rhinovirus 1A; respiratory syncytial virus; parainfluenza 1, 2, 3, 4a and 4b; and Mycoplasma pneumoniae and Streptococcus pneumoniae were analyzed [2]. These results were validated in a study where the specificity of these sets of primers and probes was evaluated, finding no cross-reactivity in the 43 clinical samples positive for these same respiratory viruses [8].

To verify the absence of cross-reactivity in the main respiratory pathogens detectable in the patients of our institution, nine positive samples were analyzed for the following respiratory viruses: rhinovirus, enterovirus, respiratory syncytial virus, adenovirus and the coronaviruses 229E and HKU1 diagnosed with the FilmArray respiratory panel. These samples were taken and processed between April and September 2021 and were stored at −80 °C. The patients from whom the sample was taken accepted that a part of their sample was to be used for the present validation by signing an informed consent form.

### 2.4. Evaluation of the Clinical Performance of the Test

Similar sensitivities and specificities were observed in subgroups by gender, age and the presence of COVID symptoms (symptomatic or asymptomatic, regardless of the time of exposure or symptoms) (Supplemental Table S2).

In this verification step, clinical samples of nasopharyngeal swabs were selected from patients diagnosed with COVID-19 via a positive result from the Seegene Allplex-SARS-CoV-2 assay commercial diagnostic PCR test. The samples were stored for up to 120 days at −80 °C and had an approximate volume of 800 µL. Samples that did not show amplification of the RNase P internal control (three samples) were considered invalid and were not considered for analysis. Because the institution’s SARS-CoV-2 diagnostic algorithm requires retesting for most samples with Cts above 35, many of these samples were excluded due to insufficient volume, with only 28 samples with Cts above 35 available for this study.

Sample size was calculated according to the guidelines for the verification of real-time PCR (RT-qPCR) molecular tests for the detection of SARS-CoV-2 from the Colombian National Institute of Health (INS) (12). To determine the number of positive samples, an expected sensitivity of 90% was considered, with a maximum permissible error in the estimate of 5% and a confidence of 95% (1.96):

$$n=\frac{\left(0.90\right)\left(1-0.90\right)\left(1.96^{2}\right)}{0.05^{2}}=138$$

To determine the number of negative samples, an expected specificity of 95% was considered, with a maximum permissible error in the estimate of 5% and a confidence of 95% (1.96):

$$n=\frac{\left(0.95\right)\left(1-0.95\right)\left(1.96^{2}\right)}{0.05^{2}}=73$$

In light of these calculations, a total of 220 samples were analyzed, of which 146 were positive and 74 were negative.

The clinical performance evaluation was derived from a 2 × 2 contingency table from which the diagnostic sensitivity, diagnostic specificity, false positive rate and false negative rate were obtained, as well as the positive predictive value (PPV), the negative predictive value (NPV) and the Kappa index [13].

To determine the usefulness of the test in different population groups, the sensitivity, specificity and Kappa index were estimated by gender, age and symptoms.

The calculation of these operating characteristics was carried out using EPIDAT 3.1 and XLSTAT [13].

To evaluate the agreement between the reference assay and the modified test with viral loads below the limit of detection, 201 clinical samples (104 with results below the limit of detection for the Allplex-SARS-CoV-2 assay and 97 with results below the limit of detection for the modified CDC assay) were assayed using both methods.

## 3. Results

### 3.1. Demographic Characteristics

The demographic and clinical characteristics of the population analyzed in this study are shown in Table 2.

### 3.2. Determination of the Analytical Limit of Detection (LoD) of the Test

**Figure 1:**
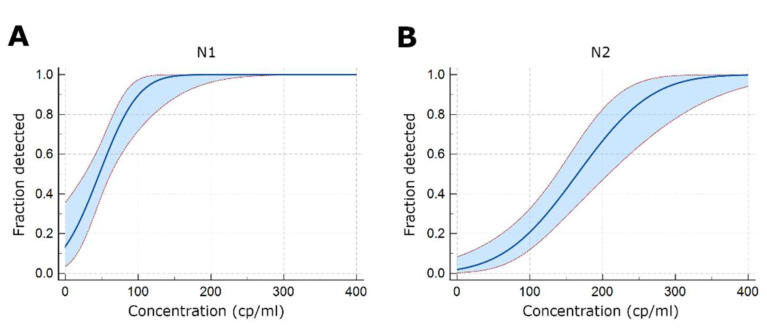
Analytical LoD of the test. (**A**) Region N1 (**B**) Region N2. Results from 20 replicates per dilution are shown. The 95% confidence interval is indicated in the light blue region.

The minimum concentration of SAR-CoV-2 that produces a positive result in 95% of the replicates was determined to be 116 copies/mL (95% CI: 91.6–189.2) for the N1 assay and 297.3 copies/mL (95% CI: 243.5–410.1) for the N2 assay (Figure 1). The standard deviation and coefficient of variance of the Ct values of 79 replicates of the positive control in separate runs (inter-assay reproducibility) were 0.47 (CV-1.3%) and 0.38 (CV-1.0%). The coefficients of variance of the Ct values of 21 assay runs with 5 replicates of spiked simulated samples (intra-assay reproducibility) were 1.1% at 400 copies/mL (*n* = 6), 1.4% at 200 copies/mL (*n* = 6), 2.1% at 100 copies/mL (*n* = 5), 1.9% at 50 copies/mL (*n* = 4), 0.2% at 25 copies/mL (*n* = 1) for the N1 target and 1.4% at 400 copies/mL (*n* = 6), 2.3% at 200 copies/mL (*n* = 6), 2.1% at 100 copies/mL (*n* = 6), 2.0% at 50 copies/mL (*n* = 1) and 1.7% at 25 copies/mL (*n* = 1) for the N2 target, for a mean inter-assay standard deviation and coefficient of variance of 0.59 (CV-1.5%) for N1 and 0.76 (CV-1.9%) for N2. When the performance of the Luna Universal Probe One-Step RT-qPCR kit was tested using NIST standards without RNA extraction, the N2 target showed a slightly lower sensitivity than the N1 target. N1 amplification was detectable in 3/3 replicates at 100.5 copies/µL, whereas the N2 amplification was detectable in 3/3 replicates at 101.5 copies/µL, but no amplification was detectable at the 100.5 copies/µL concentration, similar to what was observed with some EUA 2019-nCoV CDC assay master mixes, where several master mixes did not show amplification in 3/3 replicates at 100 copies/µL [2].

### 3.3. Determination of the Potential Cross-Reactivity of the Test

In the samples analyzed, which were found to be positive for five of the most common non-SARS-CoV-2 respiratory viruses, no amplification was observed for the N1 and N2 regions, but amplification for the RP gene was positive in eight of the nine samples (Table 3). One sample in which no amplification of the RP gene was observed was considered invalid.

### 3.4. Evaluation of the Clinical Performance of the Test

All samples from patients diagnosed with COVID-19, confirmed by RT-PCR using the Seegene Allplex-SARS-CoV-2 assay, showed amplification, while no amplification was detected for negative patients, leading to optimal operational parameters for the test. Notably, our results exclude sensitivities and specificities worse than 97.5% and 95.14%, respectively, with 95% confidence (Table 4). According to the Cohen Kappa index scale, the strength of agreement obtained between the COVID-19 diagnosis and the modified test was almost perfect [13]. Similar sensitivities and specificities were observed in subgroups by gender, age and the presence of COVID symptoms (symptomatic or asymptomatic, regardless of the time of exposure or symptoms) (Supplemental Table S2).

Five samples, all from patients with a positive COVID-19 diagnosis, amplified with Cycle Thresholds (Ct) above 37 for N1 and/or 39 for N2. These Cts correspond to samples with approximately 200 copies/mL of viral RNA and are below the detection limit of the test for the N2 target. When the Cts of these samples were compared with the Cts obtained with the Allplex-SARS-CoV-2 test (Seegene, Korea) (Table 5), it was found that all of them have amplification of at least one gene with Cts above 37, which indicates the presence of very low amounts of viral RNA and is representative of the test performance under these conditions. No samples with a negative result by the Allplex-SARS-CoV-2 method showed amplification of the N1 target or the N2 target using the evaluated method. Samples with Cts in this range should be retested to reduce the risk of false positive results.

Throughout the validation, a total of 48 negative controls were run per target (24 negative controls from the Abbott kit and 24 consisting of the viral transport medium without sample-empty VTM). No amplification was observed in any of the negative controls tested, even when these were run in wells close to positive controls or highly positive samples.

**Figure 2:**
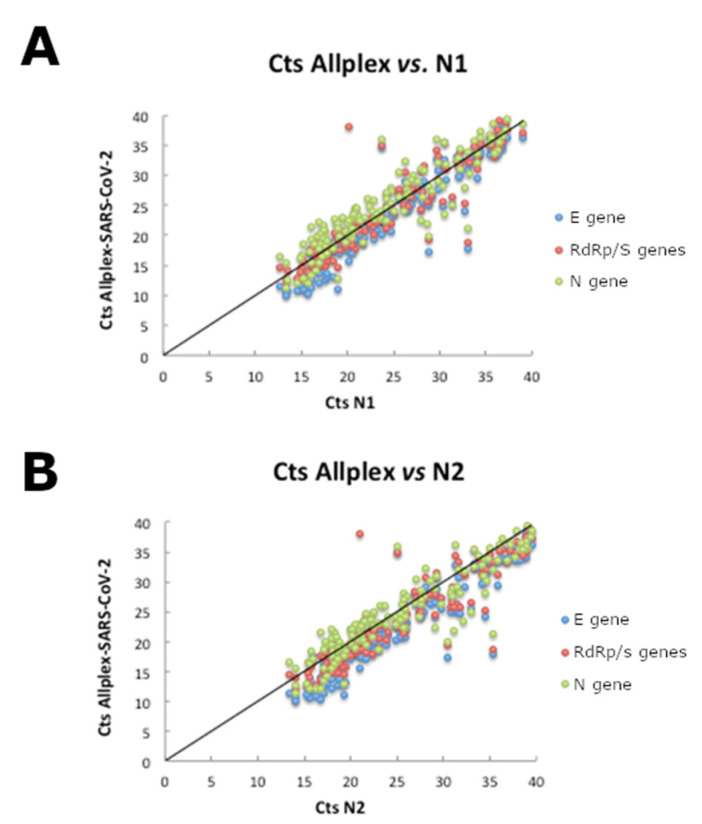
Correlation between Ct values in the CDC and Allplex tests for the N1 (**A**) and N2 (**B**) targets for the CDC assay.

Regarding the comparison of the Cts obtained with both tests analyzed, it was observed that the Cts obtained with the test based on the CDC protocol, in most cases, were highly correlated with those obtained with the Allplex-SARS-CoV-2 test. Between the N1 region and the E, RdRP/S and N genes of the Allplex-SARS-CoV-2 kit, correlations (r2) of 0.88, 0.84 and 0.82 were found, respectively (Figure 2A). Very similar correlation levels were found between the N2 region and the E, RdRP/S and N genes of the Allplex-SARS-CoV-2 kit—0.88, 0.84 and 0.82, respectively (Figure 2B). Accurate Ct values were obtained with master mixes that were frozen for at least 2 months and after two freeze-thaw cycles (data not shown).

As expected, the agreement between the two assays when testing samples with viral loads below the limit of detection was significantly lower, with 67% of the samples testing positive for both assays, 17.6% testing positive for the modified CDC assay but negative for the Allplex-SARS-CoV-2 assay and 25.8% testing positive for the Allplex-SARS-CoV-2 assay but negative for the modified CDC assay (*n* = 201). These low viral load samples are relatively rare (<5%), and the results should be interpreted with caution, since the repeated testing of these samples using the same or alternative assays may not yield consistent results. There was significant agreement between the Ct values for the samples below the limit of detection, with both assays reporting high Ct values, but a significant number of samples or specific targets for a given sample were undetectable (Supplementary Figure S1).

### 3.5. Evaluation of Different SARS-CoV-2 Variants

The processing of blinded samples from an external quality control program (QCMD) shows that the test is able to detect the B.1, B1.1.7 and B1.351 variants, demonstrating broad sensitivity (Supplemental Table S3).

## 4. Discussion

The COVID-19 pandemic has required PCR-based testing on an enormous scale, straining laboratories and institutions worldwide with unprecedented costs and logistical challenges. SARS-CoV-2 will likely continue to circulate for many years, with novel variants resulting in periodic outbreaks. For this reason, low-cost alternatives with a high diagnostic performance are required to guarantee the adequate surveillance of cases.

The implementation and validation of cost-effective diagnostic tests for COVID-19 is a necessity in Latin American countries, in which one of the problems in managing the COVID-19 pandemic is insufficient diagnostic capacity, which, for some countries, does not exceed 22% of the actual need [15].

The test is carried out in two separate assays that can each serve as a control for possible contamination in each other. Contamination during sample processing is a frequent problem that has been highlighted by alerts issued by the World Health Organization [16] and national regulatory bodies [17]. Furthermore, the test uses human RNase P as an endogenous internal control, which guarantees proper sample collection and transport and is often difficult to control in a clinical setting. Finally, the test is able to detect at least two novel variants of the virus (B1.1.7, also known as Delta, and B1.351, also known as beta), which suggests a broad spectrum of detection for the test. Finally, when compared to other commercial protocols, our assay showed important cost advantages and the availability of reagents, providing a robust and cost-efficient method for COVID-19 testing and diagnosis.

## Figures and Tables

**Table 1:** List of primer and probe sets.

Assay	Primer/Probe Name	Sequence 5′→3′
1	2019-nCov_N1 Forward	GACCCCAAAATCAGCGAAAT
	2019-nCov_N1 Reverse	TCTGGTTACTGCCAGTTGAATCTG
	2019-nCov_N1 probe	FAM-ACCCCGCATTACGTTTGGTGGACC-BHQ1
	Human Rnase P Forward	AGATTTGGACCTGCGAGCG
	Human Rnase P Reverse	GAGCGGCTGTCTCCACAAGT
	Human Rnase P Probe	Quasar 670-TTCTGACCTGAAGGCTCTGCGCG-BHQ2
2	2019-nCov_N2 Forward	TTACAAACATTGGCCGCAAA
	2019-nCov_N2 Reverse	GCGCGACATTCCGAAGAA
	2019-nCov_N2 probe	FAM-ACAATTTGCCCCCAGCGCTTCAG-BHQ1

**Table 2:** Demographic characteristics of the analyzed samples.

Variable	N (%)	Mean Age (S.D.)
**Positive cases**		
**Gender**		
Female	74 (50.7)	
Male	72 (49.3)	
**Age**		
<18 years	5 (3.4)	3 years (±5 years)
18–60 years	92 (63.0)	37 years (±12 years)
>60 years	49 (33.6)	74 years (±10 years)
**Clinical presentation**		
Symptomatic	133 (91.1)	
Asymptomatic	13 (8.9)	
**Total cases**	146	
**Negative cases**		
**Gender**		
Female	43 (58.1)	
Male	31 (41.9)	
**Age**		
<18 years	14 (18.9)	5 years (±4 years)
18–60 years	43 (58.1)	28 years (±9 years)
>60 years	17 (23.0)	75 years (±10 years)
**Clinical presentation**		
Symptomatic	54 (73)	
Asymptomatic	20 (27)	
**Total cases**	74	

**Table 3:** Results of the analysis of the cross-reactivity of the assay based on the CDC protocol against other respiratory viruses.

Sample	Respiratory Pathogen	Multiplex PCR Result	SARS-CoV-2 RT-PCR Result		
			N1	RP (Ct)	N2
1	RSV	Pos	Neg	Pos (27.30)	Neg
2	RHINO/ENTE	Pos	Neg	Pos (24.84)	Neg
3	229E	Pos	Neg	Pos (36.75)	Neg
4	RSV	Pos	Neg	Pos (25.17)	Neg
5	RHINO/ENTE	Pos	Neg	Pos (21.33)	Neg
6	HKU1	Pos	Neg	Pos (30.12)	Neg
7	RSV	Pos	Neg	Pos (27.36)	Neg
8	ADV	Pos	Neg	Pos (26.54)	Neg

RSV: Respiratory syncytial virus. RHINO/ENTE: Rhinovirus and Enterovirus. ADV: Adenovirus. Pos: positive, Neg: negative.

**Table 4:** Operational parameters of the test. PPV: Positive predictive value. NPV: Negative predictive value.

Parameter	Result	95% CI
Sensitivity (%)	100	97.50–100
Specificity (%)	100	95.14–100
False positive rate	0	
False negative rate	0	
PPV (%)	100	97.50–100
NPV (%)	100	95.14–100
Kappa Index	1	0.98–1.00

**Table 5:** Comparison of Ct values between the commercial (Allplex-SARS-CoV-2) and the modified CDC test for samples below the test’s limit of detection.

Sample	Allplex-SARS-CoV-2 Assay				Modified CDC Test		
		Target			Target		
	E	RdRP/S	N	Result	N1	N2	Result
1	36.12	37.13	38.54	Positive	39.07	39.49	Positive *
2	NA	38.72	NA	Positive	37.76	NA	Positive *
3	37.49	39.21	35.64	Positive	36.45	39.02	Positive *
4	36.17	37.65	39.42	Positive	37.33	39.03	Positive *
5	38.59	38.88	37.57	Positive	37.15	39.31	Positive *

* denotes that the results fall below the established LoD of the test and would therefore be reported as positive-below LoD.

## Data Availability

Not Applicable.

## Supplementary Materials

The following supporting information can be downloaded at: https://www.mdpi.com/article/10.3390/mps5050073/s1, Figure S1: Correlation between Ct values for the Allplex test and for the N1 target in the CDC test for samples with viral loads below the Limit of Detection; Table S1: Sample and elution volumes of commercially available RNA extraction kits and procedures that have been qualified and validated or accepted for use with the EUA 2019-nCoV CDC assay and of the Abbott m2000sp automated sample preparation system.; Table S2: Clinical sensitivity, specificity and Kappa index for the modified CDC test when applied to different population subgroups; Table S3: Ct values for detection of QCMD external quality control samples, including variants B1.1.7 and B1.351.

## References

[1] Harapan H., Itoh N., Yufika A., Winardi W., Keam S., Te H., Megawati D., Hayati Z., Wagner A.L., Mudatsir M. (2020). Coronavirus disease 2019 (COVID-19): A literature review. J. Infect. Public Health.

[2] Lu X., Wang L., Sakthivel S.K., Whitaker B., Murray J., Kamili S., Lynch B., Malapati L., Burke S.A., Harcourt J. (2020). US CDC Real-Time Reverse Transcription PCR Panel for Detection of Severe Acute Respiratory Syndrome Coronavirus 2. Emerg. Infect. Dis..

[3] Mögling R., Meijer A., Berginc N., Bruisten S., Charrel R., Coutard B., Eckerle I., Enouf V., Hungnes O., Korukluoglu G. (2020). Delayed Laboratory Response to COVID-19 Caused by Molecular Diagnostic Contamination. Emerg. Infect. Dis..

[4] Huggett J.F., Benes V., Bustin S.A., Garson J.A., Harris K., Kammel M., Kubista M., McHugh T.D., Moran-Gilad J., Nolan T. (2020). Cautionary Note on Contamination of Reagents Used for Molecular Detection of SARS-CoV-2. Clin. Chem..

[5] Cohen J. (2020). The United States badly bungled coronavirus testing—but things may soon improve. Science.

[6] Why the CDC Botched Its Coronavirus Testing|MIT Technology Review. https://www.technologyreview.com/2020/03/05/905484/why-the-cdc-botched-its-coronavirus-testing/.

[7] Vogels C.B.F., Brito A.F., Wyllie A.L., Fauver J.R., Ott I.M., Kalinich C.C., Petrone M.E., Casanovas-Massana A., Catherine Muenker M., Moore A.J. (2020). Analytical sensitivity and efficiency comparisons of SARS-CoV-2 RT–qPCR primer–probe sets. Nat. Microbiol..

[8] Nalla A.K., Casto A.M., Casto A.M., Huang M.L.W., Perchetti G.A., Sampoleo R., Shrestha L., Wei Y., Zhu H., Jerome K.R. (2020). Comparative performance of SARS-CoV-2 detection assays using seven different primer-probe sets and one assay kit. J. Clin. Microbiol..

[9] Perchetti G.A., Nalla A.K., Huang M.L., Jerome K.R., Greninger A.L. (2020). Multiplexing primer/probe sets for detection of SARS-CoV-2 by qRT-PCR. J. Clin. Virol..

[10] Lima A., Healer V., Vendrone E., Silbert S. (2020). Validation of a modified CDC assay and performance comparison with the NeuMoDxTM and DiaSorin^®^ automated assays for rapid detection of SARS-CoV-2 in respiratory specimens. J. Clin. Virol..

[11] Vogels C.B.F., Watkins A.E., Harden C.A., Brackney D., Shafer J., Wang J., Caraballo C., Kalinich C.C., Ott I., Fauver J.R. (2020). SalivaDirect: Simple and sensitive molecular diagnostic test for SARS-CoV-2 surveillance. MedRxiv.

[12] Lineamientos para la Gestión de Muestras Durante la Pandemia del SARS-CoV-2 (COVID-19) en Colombia. psps02-lineamientos-gmuestras-pandemia-sars-cov-2-col.pdf.

[13] Dir Redes en Salud Publica, D.V. y A. del R. Protocolo de Verificación (Validación Secundaria) para Pruebas Moleculares de PCR en Tiempo Real (RT-qPCR) para la Detección del SARS-CoV-2. Inst. Nac. Salud. 1–8. http://www.saludcapital.gov.co/CTDLab/Publicaciones/2021/Protocolo_verifi_RT_PCR_SARS_CoV-2.pdf.

[14] INSTRUCTION MANUAL Luna ® Universal Probe One-Step RT-qPCR Kit NEB #E3006S/L/X/E 200/500/1,000/2,500 Reactions. https://international.neb.com/-/media/nebus/files/manuals/manuale3006.pdf?rev=389c4c496bba40a2ad7e0171c37c8658&hash=B1948A5B6DDC71B2ED2DAACD2FBF93F2.

[15] Burki T. (2020). COVID-19 in Latin America. Lancet Infect. Dis..

[16] AVISO DE LA OMS PARA LOS USUARIOS DE PRODUCTOS DE DIAGNÓSTICO IN VITRO. https://www.who.int/es/news/item/20-01-2021-who-information-notice-for-ivd-users-2020-05.

[17] ALERTA SANITARIA Dirección de Dispositivos Médicos y Otras Tecnologías Invima Alerta Fuente de la Alerta: Organización Mundial de la Salud. https://app.invima.gov.co/alertas/ckfinder/userfiles/files/ALERTAS%20SANITARIAS/Dispositivos_Medicos/2020/Diciembre/Alerta%20No_%20%23194-2020%20-%20%20.pdf.

