# Supplementary material for: Performance Comparison of a Duplex Implementation of the CDC EUA 2019-nCoV Assay with the Seegene Allplex-SARS-CoV-2 Assay for the Detection of SARS-CoV-2 in Nasopharyngeal Swab Samples
Source: Methods Protoc. 2022 Sep 21;5(5):73. doi: 10.3390/mps5050073 (PMC9607526; doi:10.3390/mps5050073)
Supplement: Supplementary file 1 [file mps-05-00073-s001.zip › mps-1792979-supplementary.pdf]

Supplementary tables and figures

	Promega Maxwell® RSC 48 and Maxwell® CSC 48	Qiagen QIAamp® DSP Viral RNA Mini Kit or QIAamp® Viral RNA Mini Kit	Qiagen EZ1 Advanced XL	Roche MagNA Pure 96/24	Promega Maxwell® RSC 48 and Maxwell® CSC 48	QIAGEN QIAcube	Roche MagNA Pure LC	Roche MagNA Pure Compact	bioMérieux NucliSENS® easyMAG® Instrument	bioMérieux EMAG® Instrument	Abbott m2000sp automated Sample Preparation System
Sample Volume (ml)	120	100 or 140	120	100	120	140	100	100	100	100	600
Elution volume (ml)	75	100 or 140	120	100	75	100	100	100	100	100	80
Concentration factor	1,6	1	1	1	1,6	1,4	1	1	1	1	7,5

Table S1. Sample and elution volumes of commercially available RNA extraction kits and procedures that have been qualified and validated or accepted for use with the EUA 2019-nCoV CDC assay and of the Abbott m2000sp automated sample preparation system. Data from CDC 2019-Novel Coronavirus (2019-nCoV) Real-Time RT-PCR Diagnostic Panel For Emergency Use Only Instructions for Use https://www.fda.gov/media/134922/download.

Population subgroup	N	Sensitivity (%) (IC 95%)	Specificity (%) (IC 95%)	Kappa (IC 95%)
Women	117	100<br>(95.14 – 100)	100<br>(91.78 – 100)	1<br>(0.97 – 1.00)
Men	103	100<br>(95.01 – 100)	100<br>(88.78 - 100)	1<br>(0.96 – 1.00)
Children (<18yo)	19	100<br>(47.82 – 100)	100<br>(76.84 – 100)	1<br>(0.82 – 1.00)
18 – 60 yo	135	100<br>(96.07 – 100)	100<br>(91.78 – 100)	1<br>(0.97 – 1.00)
>60 yo	66	100<br>(92.75 – 100)	100<br>(80.49 – 100)	1<br>(0.95 – 1.00)
Symptomatic	187	100<br>(97.26 – 100)	100<br>(93.40 – 100)	1<br>(0.98 – 1.00)
Asymptomatic	33	100<br>(75.29 – 100)	100<br>(83.16 – 100)	1<br>(0.89 – 1.00)

Table S2. Clinical sensitivity, specificity and Kappa index for the modified CDC test when applied to different population subgroups.

Sample Code	Sample Content	Sample concentration	Cycle Threshold
SCV2_21C1C-01	SARS-CoV-2 Variant B.1	3.44 dPCR Log10 IU/ml	36,53
SCV2_21C1C-02	SARS-CoV-2 Variant B.1	4.05 dPCR Log10 IU/ml	33,51
SCV2_21C1C-03	SARS-CoV-2 UK (Alpha) Variant B1.1.7	4.08 dPCR Log10 IU/ml	33,1
SCV2_21C1C-04	SARS-CoV-2 SA (Beta) Variant B1.351	4.18 dPCR Log10 IU/ml	33,77
SCV2_21C1C-05	SARS-CoV-2 Variant B.1	4.92 dPCR Log10 IU/ml	30,07

Table S3. Ct values for detection of QCMD external quality control samples, including variants B1.1.7 and B1.351.

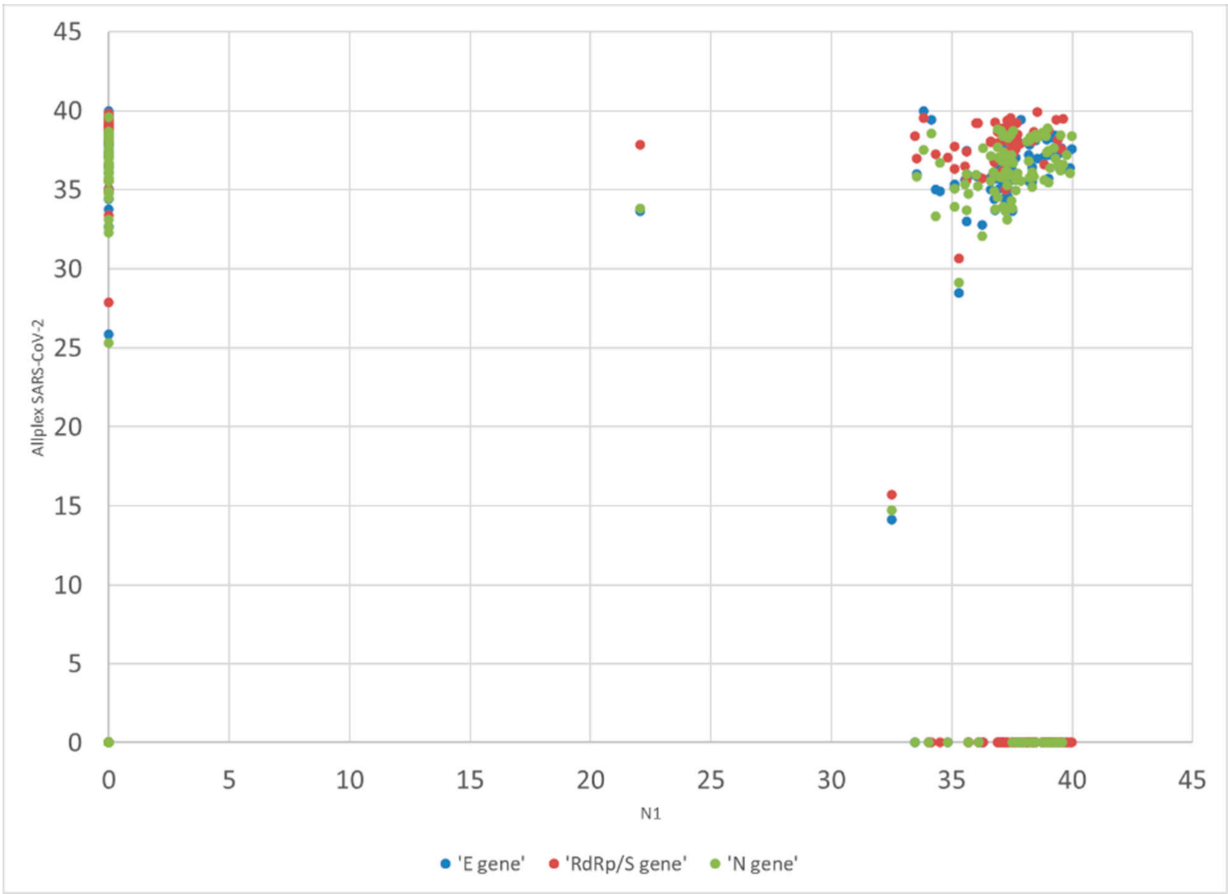

Figure S1. Correlation between Ct values for the Allplex test and for the N1 target in the CDC test for samples with viral loads below the Limit of Detection.
